# Reduction of microRNA-184 by E6 oncoprotein confers cisplatin resistance in lung cancer via increasing Bcl-2

**DOI:** 10.18632/oncotarget.8708

**Published:** 2016-04-12

**Authors:** Min-Che Tung, Po-Lin Lin, Ya-Wen Cheng, De-Wei Wu, Sauh-Der Yeh, Chi-Yi Chen, Huei Lee

**Affiliations:** ^1^ Graduate Institute of Clinical Medicine, Taipei Medical University, Taipei, Taiwan; ^2^ Graduate Institute of Cancer Biology and Drug Discovery, Taipei Medical University, Taipei, Taiwan; ^3^ Department of Surgery, Tung's Taichung Metro-Harbor Hospital, Taichung, Taiwan; ^4^ Institute of Medicine, Chung Shan Medical University, Taichung, Taiwan; ^5^ Department of Surgery, Chung Shan Medical University, Taichung, Taiwan

**Keywords:** miR-184, HPV, cisplatin resistance

## Abstract

MicroRNA-184 suppresses cell growth and survival via targeting c-Myc and Bcl- 2. We recently reported that miR-184 promotes tumor progression in non-small cell lung cancer via targeting CDC25A and c-Myc. We here hypothesized that miR-184 could be down-regulated by E6 oncoprotein to confer cisplatin resistance in NSCLC. Human papillomavirus (HPV) 16-positive lung cancer TL-1 and cervical cancer SiHa cells compared with HPV16-negative TL-10 and C33A cells were enrolled for E6 manipulation. MiR-184 expression levels were increased by E6-knockdown in TL-1 and SiHa cells, but decreased by E6-overexpression in TL-10 and C33A cells. The MTT assay showed that the inhibition concentration of cisplatin yielding for 50% cell viability was dependent on miR-184 levels. Bcl-2 de-targeted by E6-mediated miR- 184 reduction was responsible for cisplatin resistance. Luciferase reporter assay and real- time PCR analysis indicated that the miR-184 promoter activity and its expression were modulated by E6 and/or p53 manipulation. Chromatin immunoprecipitation (ChIP) assay confirmed that p53 was bound onto the miR-184 promoter and its binding activity was modulated by E6 and/or p53 manipulation. Among patients, high miR184 and high Bcl-2 mRNA expression was more commonly occurred in E6- positive tumors than in E6-negative tumors. Fifty-nine out of 136 patients receiving cisplatin-based chemotherapy were available for the retrospective study. Patients with low-mR-184, E6-positive, high-Bcl-2 tumors, and both combinations were more prevalently occurred unfavorable response to cisplatin-based chemotherapy than their counterparts. In conclusion, a decrease in miR-184 level by E6 oncoprotein may predict unfavorable response to cisplatin-based chemotherapy in HPV-infected NSCLC patients via increasing Bcl-2 expression.

## INTRODUCTION

MicroRNA-184 (miR-184) plays a dual role in human cancers. For example, miR-184 inhibits cell growth and invasion capability in glioma and neuroblastoma cells [1, 2]. However, miR-184 plays an oncogenic role in tongue squamous cell carcinoma [3]. MiR-184 directly targets c-Myc to suppress cell growth in non-small cell lung cancer (NSCLC) [4]. We recently reported that a decrease in miR-184 by miR-21 promotes tumor malignancy and poor outcomes in non-small cell lung cancer (NSCLC) via targeting CDC25A and c-Myc [5]. Therefore, miR-184 might play a tumor suppressor role in NSCLC.

Our previous studies indicated that human papillomavirus (HPV) 16/18 may be associated with the development of NSCLC in Taiwan and promotes tumor malignancy via increasing human telomerase reverse transcriptase, FoxM1, IL-10 expressions, and inactivation of p53 and TIMP-3 by E6 oncoprotein [6–10]. However, the involvement of HPV in lung tumorigenesis is still controversial. This conflicting could be due to the geographic variation [10–15]. To elucidate which miRs could be linked with HPV-associated lung tumorigenesis, miR microarray analysis showed that miR-184 expression levels increased ~14-fold in E6-knockdown TL-1 cells when compared with TL-1 cells transfected with non-specific small hairpin RNA (NC). Bcl-2 plays a central role in resistance to apoptosis [16–18], and its expression can be down-regulated by miR-184 [19]. A previous study indicated that miR-184 levels in H1299 cells can be elevated by ectopic wild-type p53 expression [20]. We therefore hypothesized that a decrease in miR-184 expression due to p53 degradation by E6 oncoprotein may confer cisplatin resistance in NSCLC via increasing Bcl-2 expression.

## RESULTS

### A decrease in miR-184 expression by E6 oncoprotein confers cisplatin resistance

**Figure 1:**
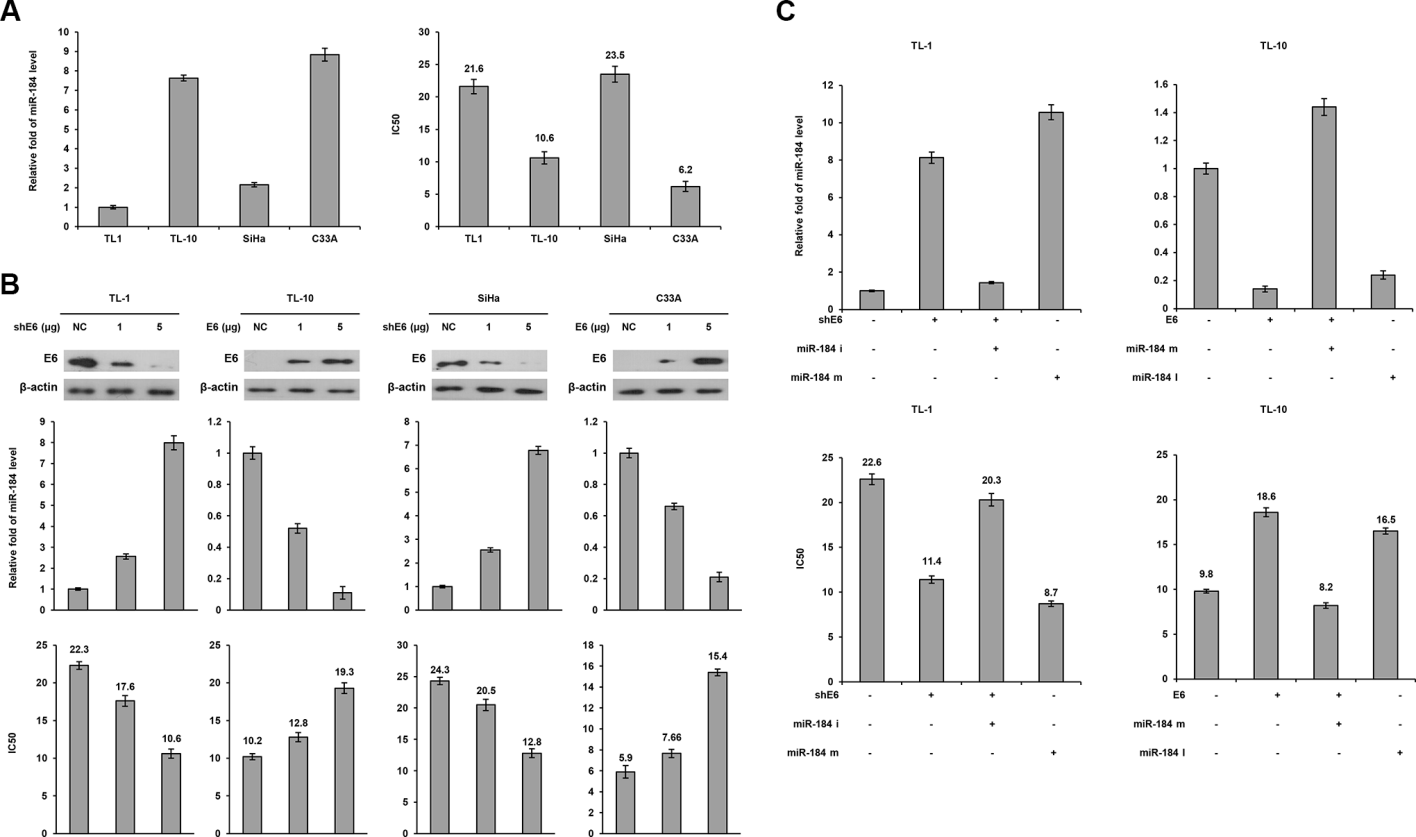
A decrease in MiR-184 level by E6 oncoprotein confers cisplatin resistance (**A**) Expression levels of miR-184 were evaluated by real-time PCR in four cancer cell lines. The cell viability was determined by the MTT assay after the Cells were treated with or without cisplatin (0, 2, 4, 8, 16, 32 μM) treatment for 48 h for calculation of the IC50 value. (**B**) shE6 plasmids were transfected into E6-positive cell lines (TL-1 and SiHa) compared with both cell types transfected with a non-specific shRNA (NC), E6 expression vector were transfected into E6 negative (TL-10 and C33A) cell lines compared with both cell types transfected with an empty vector (VC). After 24 h, the indicated cells were incubated with or without cisplatin (0, 2, 4, 8, 16, 32 μM) for 48 h and then the change of IC50 value by E6 manipulation was determined by the MTT assay. The cell lysates were separated by SDS-PAGE for the evaluation of E6 expression by specific antibodies using western blotting. The miR-184 level was determined by real-time PCR. (**C**) TL-1 and SiHa cells were treated with shE6 shRNA (5 μg) and/or miR-184 mimic (m, 40 nM). TL-10 and C33A cells were treated with E6 expression vector (5 μg) and/or miR-184 inhibitor (i, 40 nM). After 24 h, the indicated cells were incubated with or without cisplatin (0, 2, 4, 8, 16, 32 μM) for 48 h and the change of IC50 value by E6 manipulation and/or miR mimic or inhibitor was determined by the MTT assay.

HPV16-positive TL-1 and –negative TL-10 cells were enrolled to examine whether miR-184 expression in lung cancer could be up-regulated by E6 oncoprotein. HPV16-positive SiHa and –negative C33A cervical cancer cells were used as positive and negative controls. Real-time PCR analysis indicated that miR-184 expression levels were significantly lower in HPV-positive TL-1 and SiHa cells than in HPV-negative TL-10 and C33A cells (Figure 1A left panel). The MTT assay showed that the inhibition concentration of cisplatin for yielding 50% viability (IC50) was significantly higher in TL-1 cells than in TL-10 cells (21.6 vs. 10.6). A similar finding in the IC50 value for cisplatin was observed in SiHa versus C33A cells (23.5 vs. 6.2; Figure 1A right panel).

We next examined whether E6 could reduce miR- 184 expression and, in turn, confer cisplatin resistance in E6-positive cells. E6 manipulation by transfecting its shRNA and expression vector was conducted in TL-1, SiHa, TL-10 and C33A cells. Western blotting indicated that E6 expression decreased in E6-knockdown TL-1 and SiHa cells, but increased in E6-overexpressing TL-10 and C33A cells (Figure 1B upper panel). Concomitantly, miR- 184 expression levels increased in E6-knockdown TL-1 and SiHa cells, but decreased in E6-overexpressing TL-10 and C33A cells (Figure 1B middle panel). The IC50 value for cisplatin was dependent on miR-184 expression levels in these four cell types subjected to E6 manipulation (Figure 1B bottom panel). We further used miR-184 inhibitor or mimic to verify whether a decrease in miR- 184 expression by E6 could be responsible for cisplatin resistance. Real- time PCR analysis indicated that miR- 184 expression level was increased by E6-knockdown, but the increase of miR-184 expression by E6-knockdown was restored by transfecting miR-184 inhibitor in TL-1 cells (Figure 1C left upper panel). Conversely, miR-184 expression was decreased by E6 overexpression, but the decrease of miR-184 by E6 overexpression was reversed by transfecting miR-184 mimic in TL-10 cells (Figure 1C left upper panel). The IC50 value was decreased and increased concomitantly by E6 manipulation in both cell types, and the change of the IC50 value by E6 manipulation can be reversed by miR-184 inhibitor or mimic transfections (Figure 1C lower panel). The change in the IC50value by miR-184 inhibitor and mimic was relatively correspondent with the effects of E6 manipulation on the IC50 value in both cell types. These results clearly indicated that a decrease in miR-184 expression by E6 may be responsible for cisplatin resistance in NSCLC cells.

### MiR-184 transcription is down-regulated by E6 via decreased p53 binding to the miR-184 promoter due to p53 degradation by E6

**Figure 2:**
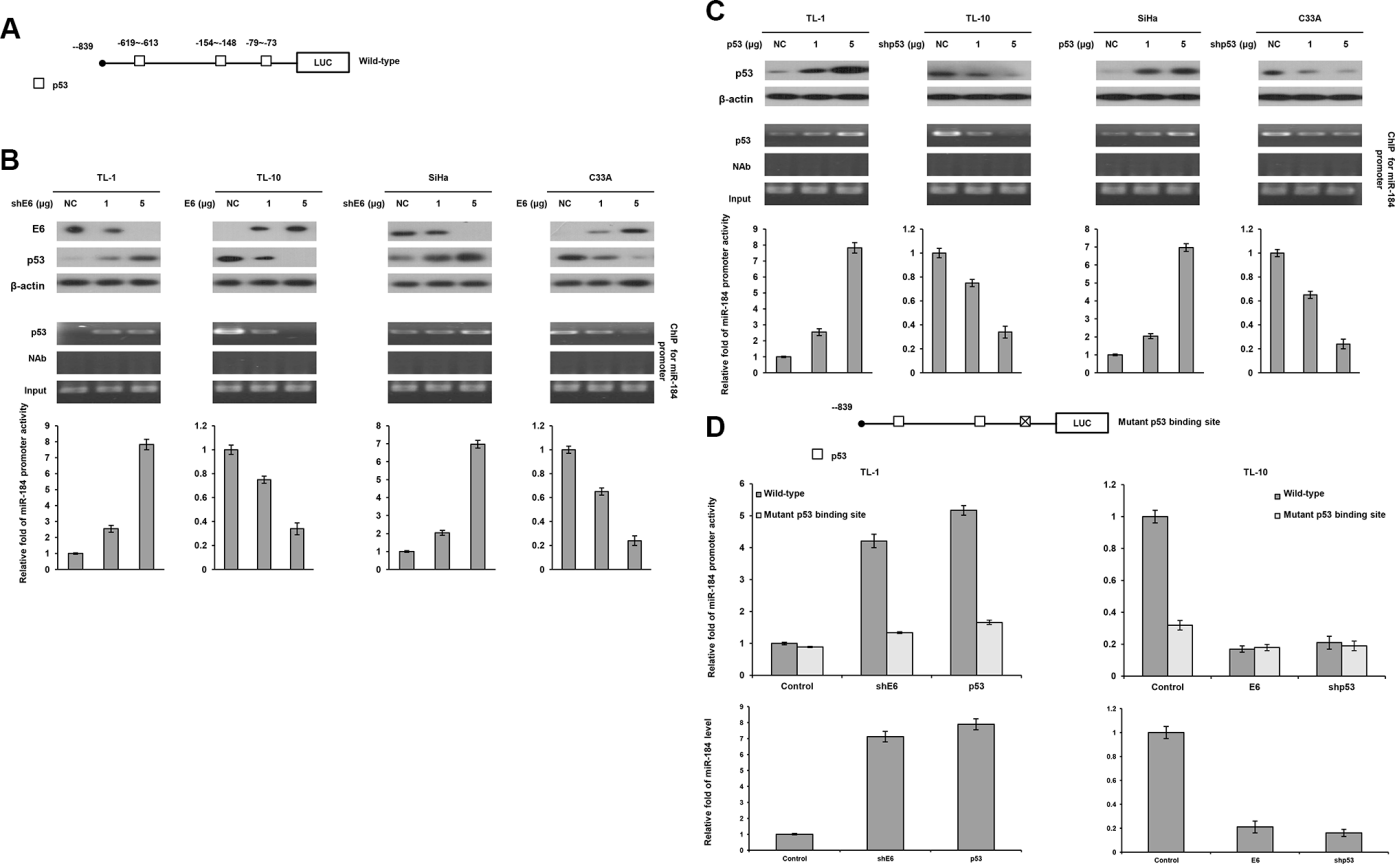
The miR-184 promoter activity is directly regulated by wild-type p53 (**A**) Diagram summarizing the positions of the p53 putative binding sites on the miR-184 promoter constructs predicted by a software analysis. (**B**) shE6 plasmids were transfected into E6-positive TL-1 and SiHa cells compared with those transfecting a non-specific shRNA (NC). On the other hand, E6 expression vector were transfected into E6-negative TL-10 and C33A cells compared with those transfecting an empty expression vector (VC). The cell lysates were separated by SDS-PAGE for the evaluation of E6 expression by a specific antibody using western blotting. A ChIP assay was performed to evaluate the binding ability of p53 onto the miR-184 promoter. The products were amplified by PCR. Luciferase reporter assay was performed to evaluate the miR-184 promoter activity in both cell types by transfecting the miR-184 promoter (−839/+1). (**C**) shE6 plasmids were transfected into E6-positive TL-1 and SiHa cells compared with their NC cells. On other hand, the shp53 were transfected into E6-negative TL-10 and C33A cells compared with their VC cells. The cell lysates were separated by SDS-PAGE for the evaluation of protein expression by specific antibodies using western blotting. A ChIP assay was performed to evaluate the binding ability of p53 onto the miR-184 promoter. The products were amplified by PCR. Luciferase reporter assay was performed to evaluate the miR-184 promoter activity in both cell types by transfecting the miR-184 promoter (−839/+1). (**D**) The miR-184 promoter (−839/+1) containing wild-type- or mutant p53-binding sites were transfected into TL-10 and C33A cells and then co-transfected with wild-type p53 (5 μg), E6 expression vectors (5 μg), shp53 (5 μg) and/or shE6 shRNA (5 μg). Luciferase reporter assay was performed to evaluate the reporter activity of miR-184 promoter (−839/+1). The miR-184 level was determined by real-time PCR.

We examined the possibility that a decrease in miR-184 expression by E6 could be through deregulating miR-184 transcription due to p53 degradation by E6. This hypothesis was raised by a software analysis (http://alggen.lsi.upc.es/cgi-bin/promo_v3/promo/promoinit.cgi?dirDB=TF_8.3), and indicated that four p53 putative binding sites were existed on the miR-184 promoter (Figure 2A). The four cell types (TL-1, TL-10, SiHa, and C33A) were enrolled to transfect with shE6 or E6 expression vector. Western blotting indicated that p53 expression increased in E6-knockdown TL-1 and SiHa cells, but decreased in E6-overexpressing TL-10 and C33A cells (Figure 2B upper panel). Luciferase reporter assay indicated that the miR-184 promoter activity (−839/+1) was dose-dependently increased by E6 knockdown in TL-1 and SiHa cells, but decreased by E6 overexpression in TL-10 and C33A cells (Figure 2B lower panel). Chromatin immunoprecipitation (ChIP) assay confirmed that the binding activity of p53 onto its putative binding site of the miR-184 promoter increased in E6-knockdown TL-1 and SiHa cells, but decreased in E6-overexpressing TL-10 and C33A cells in a dose-dependent manner (Figure 2B middle panel).

We next examined whether p53 could directly regulate miR-184 expression, the four cell types were transfected with p53 expression vector or shp53. Western blotting indicated that p53 expression increased in p53-overexpressing TL-1 and SiHa cells, but decreased in p53-knockdown TL-10 and C33A cells (Figure 2C upper panel). Luciferase reporter assay indicated that the miR-184 promoter activity (−839/+1) was significantly increased by p53 overexpression in TL-1 and SiHa cells, but decreased by p53 knockdown in TL-10 and C33A cells (Figure 2C lower panel). ChIP assay confirmed that the binding activity of p53 onto its putative binding site of the miR-184 promoter was markedly increased by p53 expression vector transfection in TL-1 and SiHa cells, but decreased by p53 knockdown in TL-10 and C33A cells (Figure 2C middle panel).

We further constructed a miR-184 promoter harboring a p53 mutated binding site near the transcription start site by site-directed mutagenesis (Figure 2D). Luciferase reporter assay indicated that the miR-184 promoter activity and miR-184 expression levels in TL-1 cells with the wild-type miR-184 promoter transfection were markedly elevated by co-transfecting shE6 or p53 expression vector, but the increase of miR-184 promoter activity and miR-184 expression levels by both treatments were restored by transfecting the mutant miR-184 promoter (Figure 2D). On the other hand, the miR-184 promoter activity and miR-184 expression levels were significantly decreased by transfecting the mutant miR-184 promoter compared with transfecting the wild-type miR-184 in TL-10 cells, but the miR-184 promoter activity and miR-184 expression levels were rescued by E6 or shp53 transfection in TL-10 cells with the wild-type or the mutant miR-184 promoter transfection (Figure 2D).

**Figure 3:**
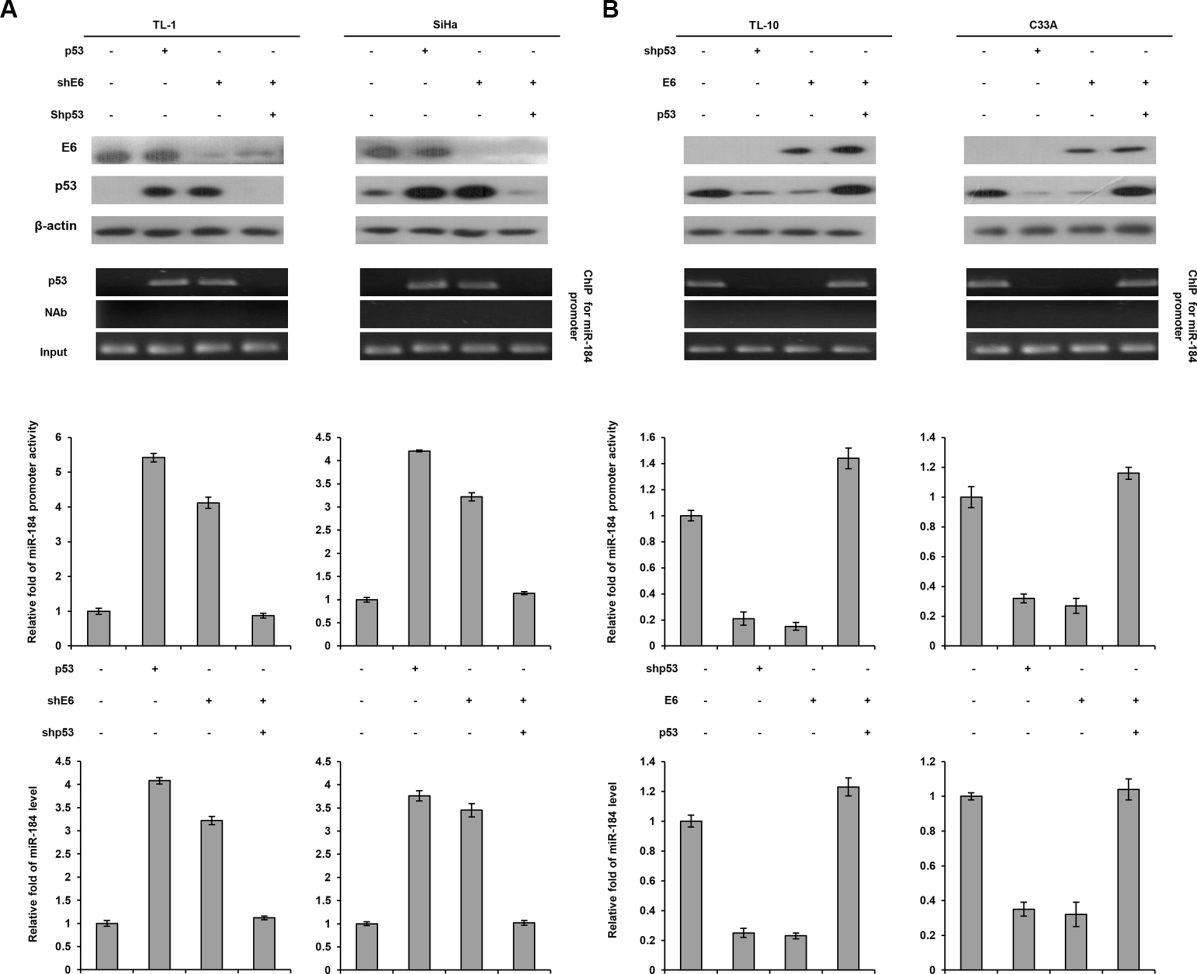
MiR-184 expression is down-regulated by E6 oncoprotein at transcription level via decreased the binding activity of p53 onto the miR-184 promoter due to p53 degradation by E6 (**A**) TL-1 and SiHa cells were treated with miR- 184 promoter reporter plasmid (−839/+1), wild-type p53 expression vector (5 μg), shp53 (5 μg), and/or shE6 shRNA (5 μg). (**B**) TL-10 and C33A were treated with the miR-184 promoter reporter plasmid (−839/+1), shp53 shRNA (5 μg), wild-type p53 (5 μg), and/or E6 expression vectors (5 μg). The cell lysates were separated by SDS-PAGE for the evaluation of protein expression by specific antibodies using western blotting. A ChIP assay was performed to evaluate the binding ability of p53 onto the miR-184 promoter. The products were amplified by PCR. Luciferase reporter assay was performed to evaluate the reporter activity of miR-184 promoter (−839/+1). The miR-184 level was determined by real-time PCR.

The four cell types were further enrolled to transfect with p53 expression vector, shp53, and/or shE6. Western blotting indicated that p53 expression level was increased by p53 overexpression, but decreased by E6 knockdown in TL-1 cells. The change of the p53 level by p53 overexpression or E6 silencing can be rescued by shE6 + shp53 transfection in TL-1 cells when compared with NC cells (Figure 3A left panel). Luciferase reporter assay indicated that the miR- 184 promoter activity (−839/+1) was increased by p53 overexpression, but decreased by E6 knockdown in TL-1 cells. The change of the miR-184 promoter activity by p53 overexpression or E6 silencing can be rescued by shE6 + shp53 transfection in TL-1 cells (Figure 3A left panel). The change of the miR-184 promoter activity was consistent with miR-184 expression in TL-1 cells subjected to the same treatments (Figure 3A left panel). ChIP assay further indicated that p53 bound to its putative binding site of the miR-184 promoter in TL-1 cells transfecting p53 expression vector or shE6. However, the p53 binding to the miR-184 promoter in TL-1 cells was disappeared by shE6 + shp53 transfection (Figure 3A left bottom panel). Similar findings in the miR-184 promoter activity, miR-184 expression, and the binding activity of p53 onto the miR-184 promoter were revealed in SiHa cells subjected to the same treatments (Figure 3A right panel). On the other hand, HPV-negative TL-10 and C33A cells were transfected with shp53, p53, and/or E6 expression vector to verify whether miR-184 expression was down-regulated by E6 via decreased p53 binding to the miR-184 promoter. The miR-184 promoter, miR-184 expression, and the binding activity of p53 onto the miR-184 promoter were decreased by E6 overexpression (Figure 3B). We therefore suggest that miR-184 is directly up-regulated by wild-type p53 at transcription level.

**Figure 4:**
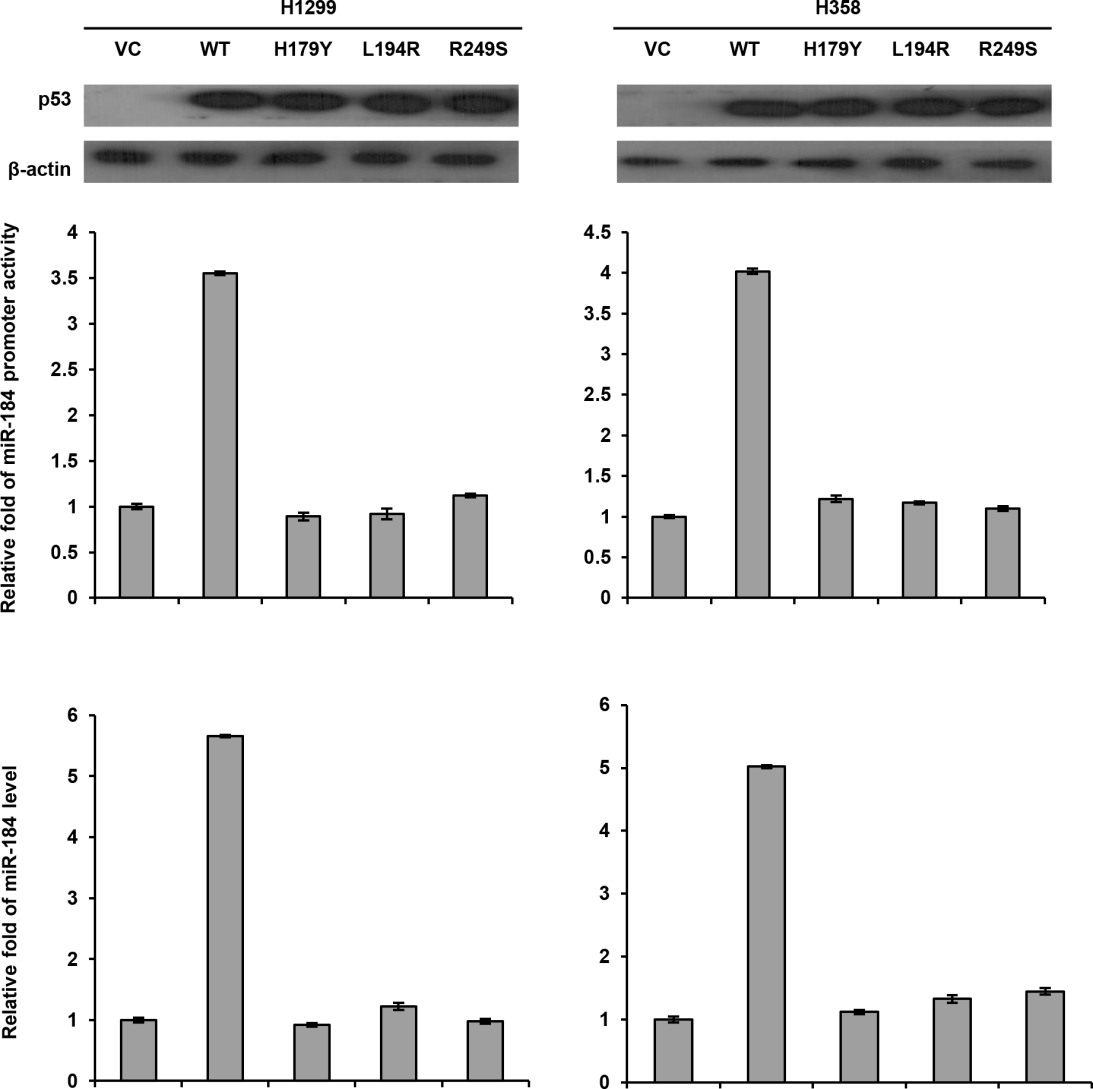
The miR-184 promoter activity is dependent on p53 status A wild-type p53 or different mutant p53 expression vectors (5 μg) and the miR-184 promoter reporter plasmid were transfected into H1299 and H358 cells and the then cells lysates were separated by SDS-PAGE for the evaluating p53 expression by western blotting using a specific antibody. Luciferase reporter assay was performed to evaluate the reporter activity of miR-184 promoter (−839/+1). The miR-184 level was determined by real-time PCR.

Next, we transfected the wild-type or different mutant p53 expression vectors into p53-null H1299 and H358 cells to examine whether miR-184 expression could be dependent on p53 mutation status. The miR-184 promoter activity (−839/+1) and miR-184 expression levels were markedly increased by the wild-type p53 expression vector, but unchanged by the mutant p53 expression vector transfections in both cell types when compared with their VC cells (Figure 4). In addition, the miR-184 promoter activity and its expression levels were not influenced by three different p53 mutant transfections (H179Y, L194R, and R249S) when compared with their VC cells. These results clearly indicated that miR-184 expression is down-regulated by E6 at transcription level via decreased p53 binding to the miR-184 promoter due to p53 degradation by E6.

### A decrease in miR-184 expression by E6 may confer cisplatin resistance via increasing Bcl-2 expression

**Figure 5:**
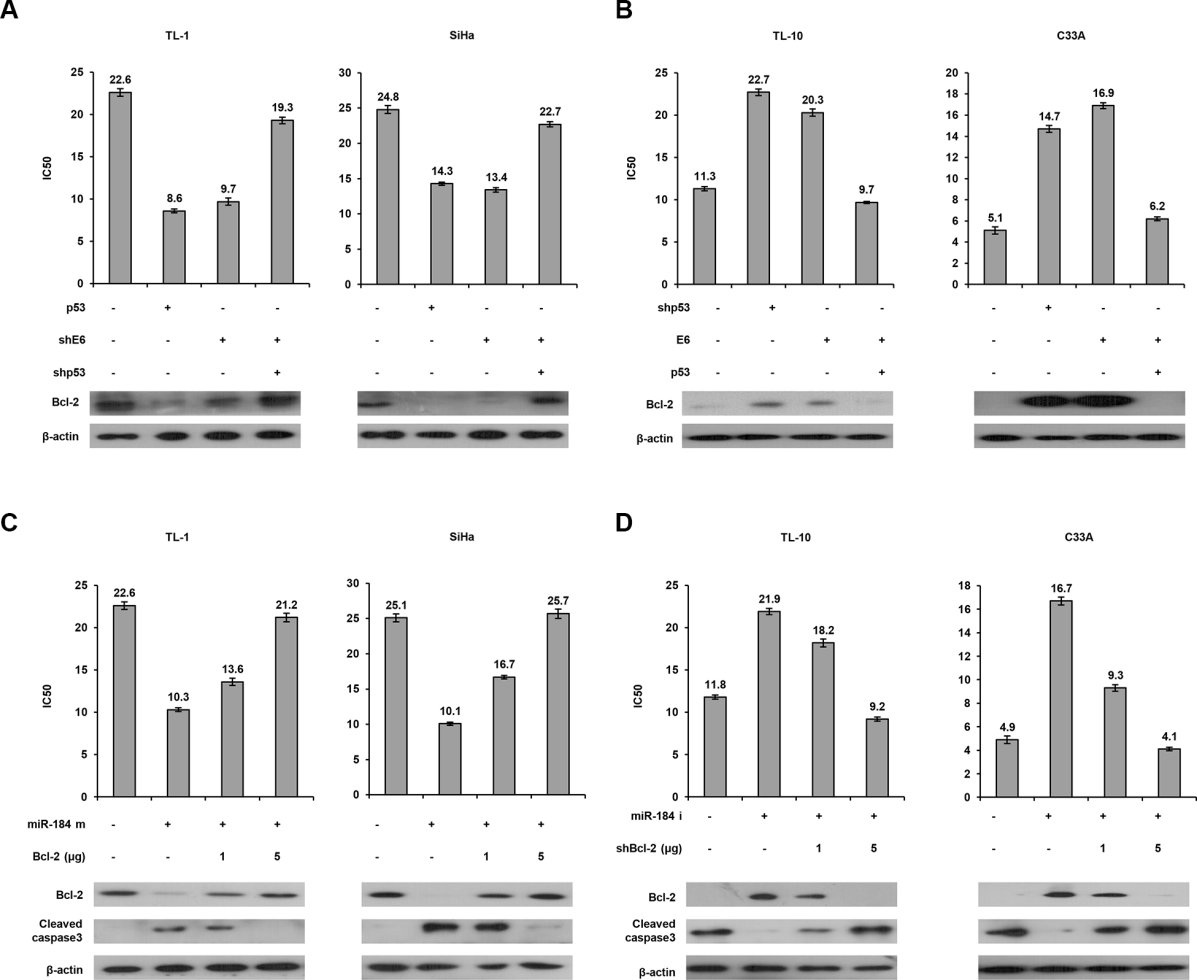
A decrease in miR-184 expression by E6 confers cisplatin resistance via increasing Bcl-2 expression (**A**) TL-1 and SiHa cells were treated with wild-type p53 expression vector (5 μg), shp53 (5 μg), and/or shE6 shRNA (5 μg). (**B**) TL-10 and C33A were treated with shp53 shRNA (5 μg), wild-type p53 (5 μg), and/or E6 expression vectors (5 μg). After 24 h, the indicated cells were treated with or without cisplatin (0, 2, 4, 8, 16, 32 μM) for 48 h and then the MTT assay was used to determine the IC50 value for cisplatin. (**C**) TL-1 and SiHa cells were treated with miR-184 mimic (m, 40 nM) and/or Bcl-2 expression vectors. (**D**) TL-10 and C33A were treated with miR-184 inhibitor (i, 40 nM) and/or shBcl-2. After 24 h, the indicated cells were incubated with or without cisplatin (0, 2, 4, 8, 16, 32 μM) for 48 h and the IC50 value of both cell types transfected with miR-184 inhibitor and/or shBcl-2 was determined by the MTT assay. The cell lysates were separated by SDS-PAGE for the evaluation the expression of Bcl-2 and cleaved caspase3 by western blotting using their specific antibodies.

MiR-184 suppresses cell growth and survival in nasopharyngeal cancer via targeting Bcl-2 [19]. We therefore examined the possibility that a decrease in miR-184 by E6 may confer cisplatin resistance due to de- targeting Bcl-2. The MTT assay indicated that the IC50 value was markedly decreased by p53 overexpression or E6 knockdown, but the decrease of the IC50 value by p53 overexpression or E6 knockdown can be reversed by shE6 + shp53 transfection in TL-1 cells (Figure 5A left upper panel). Western blotting indicated that Bcl-2 expression was concomitantly decreased by p53 overexpression or E6 knockdown in TL-1 cells, but the decrease of Bcl-2 expression by both treatments can be rescued by shE6 + shp53 transfection in TL-1 cells compared with NC cells (Figure 5A left lower panel). Similar findings in Bcl-2 were observed in SiHa cells subjected to the same treatments (Figure 5A right panel). On the other hand, TL- 10 and C33A cells were transfected with shp53, p53, and/or E6 expression vector. The IC50 value was increased by shp53 or E6 transfection, but the increase of the IC50 value by both treatments was reversed by E6 + p53 transfection in TL-10 and C33A cells (Figure 5B upper panel). Similarly, Bcl-2 expression was concomitantly increased by shp53 or E6 transfection, but the increase of Bcl-2 expression by both treatments was rescued by E6 + p53 transfection in both cell types (Figure 5B lower panel).

**Figure 6:**
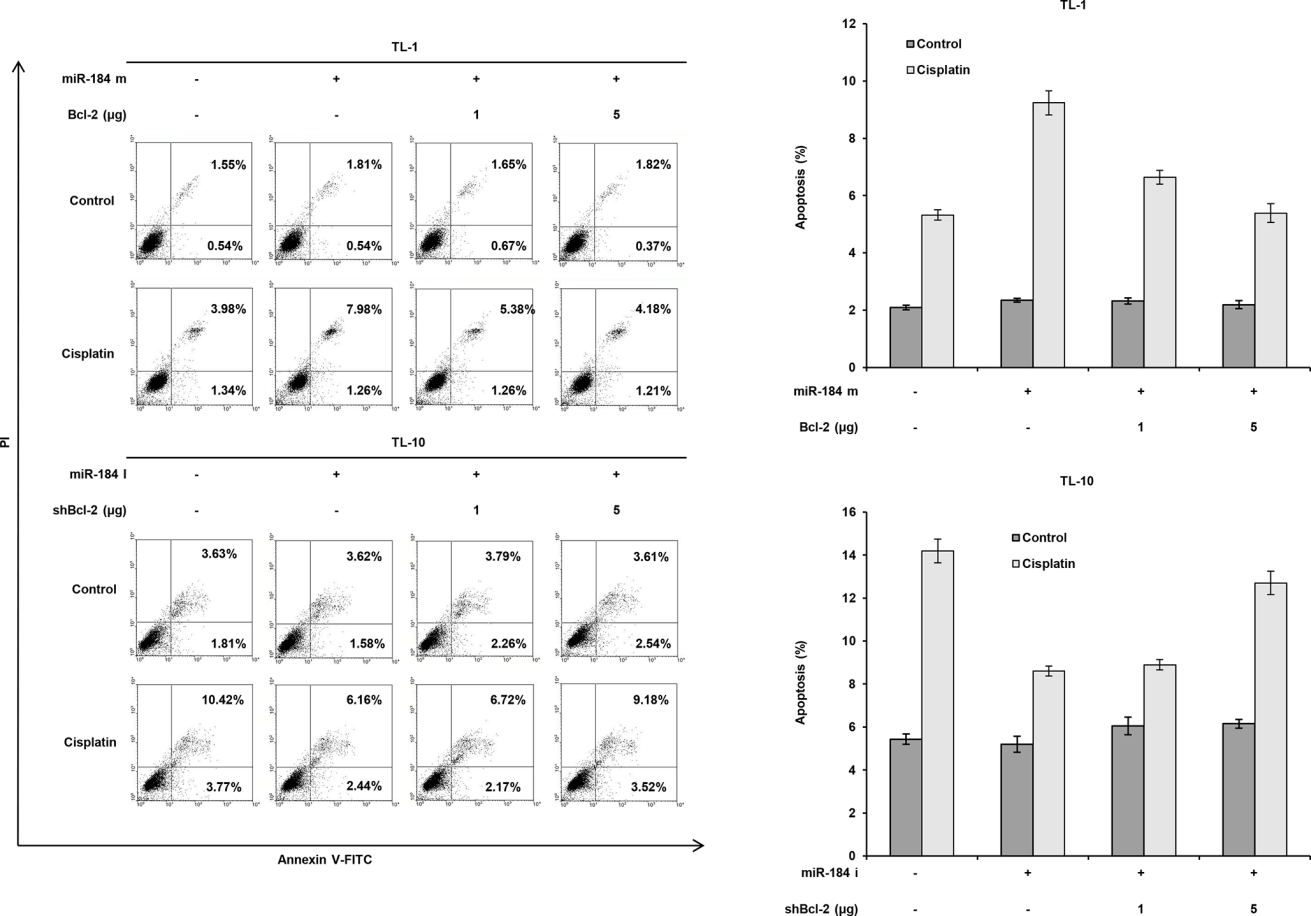
Increase in Bcl-2 expression by reduced miR-184 may be responsible for cisplatin resistance via apoptotic pathway TL-1 cells were treated with miR-184 mimic (m, 40 nM) and/or Bcl-2 expression vectors. TL-10 cells were treated with miR- 184 inhibitor (miR-184i, 40 nM) and/or shBcl-2 for 24 h. Indicated cells were treated with 0.1% DMSO or 5 μM cisplatin for 24 h. The cells were subjected to annexin V and PI staining by a flow cytometry analysis. Percentage of apoptotic cells including with the Annexin V+/PI- population (early apoptosis) plus Annexin V+/PI− (late apoptosis/secondary necrosis) was summarized by a flow cytometric analysis. Data are expressed as means ± s.d. (*n* = 3).

To verify whether Bcl-2 de-targeted by E6- mediated miR-184 reduction could be responsible for cisplatin resistance. TL-1 and SiHa cells were transfected with miR- 184 mimic and/or co-transfected with Bcl- 2 expression vector. The IC50 value was concomitantly decreased by transfecting miR-184 mimic, but was gradually increased by Bcl-2 expression vector co- transfection in TL-1 and SiHa cells (Figure 5D upper panel). Bcl-2 expression levels were significantly reduced by transfecting miR-184 mimic, but the Bcl- 2 expression levels were dose-dependently elevated by transfecting Bcl- 2 expression vector in both cell types (Figure 5C lower panel). On the other hand, the IC50 value was elevated by the increase of Bcl-2 expression due to miR- 184 inhibitor transfection, but the IC50 value was reduced by Bcl-2 silencing in TL-10 and C33A cells (Figure 5D upper panel). The change of the IC50 value by miR-184 mimic and/or Bcl- 2 silencing in TL- 10 and C33A cells was dependent on Bcl- 2 expression (Figure 5D lower panel). In addition, the expression of cleaved caspase 3 was increased by miR-184 mimic transfection, but was nearly completely rescued by Bcl- 2 overexpression in TL-1 and SiHa cells. The opposite in the expression of cleaved caspase 3 was revealed in TL-10 and C33A transfecting miR-184 inhibitor and/or shBcl-2 (Figure 5C lower panel). We further examined whether apoptotic pathway could be involved in miR-184-mediated cell death, a flow cytometry analysis coupling with PI staining was performed in TL-1 and TL-10 cells (Figure 6 left panel). Data showed that that the percentage of apoptotic cells was significantly increased by miR-184 mimic in TL-1 cells, but the increase of apoptotic cells was decreased by Bcl2 co-transfection in a dose dependent manner (Figure 6 right upper panel). The opposite in the percentage of apoptotic cells was observed in TL-10 cells transfecting with miR-184 inhibitor and/or shBcl-2 (Figure 6 right lower panel). These results suggest that an increase in Bcl-2 expression by reduced miR-184 may be responsible for E6-mediated cisplatin resistance in NSCLC cells via apoptotic pathway.

### Low miR-184 and high Bcl-2 mRNA expression are more commonly occurred in E6-positive tumors than in E6-negative tumors

We enrolled 136 tumors from NSCLC patients to examine the possibility that E6 could be correlated with Bcl-2 mRNA and miR-184 expression and miR-184 could be associated with Bcl-2 mRNA levels in lung tumors. The immunostaining results of E6 oncoprotein in lung tumors were obtained from our previous reports [6–11]. MiR- 184 and Bcl-2 mRNA expression in lung tumors were evaluated by real-time PCR analysis. The median value of miR-184 and Bcl-2 mRNA levels was used as a cutoff point to divide patients into “high” and “low” subgroup. As shown in Table 1, high Bcl-2 mRNA expression was marginally correlated with E6 expression in lung tumors (50% vs. 33.8%, *P* = 0.056). Low miR-184 expression was more frequently observed in E6-positive tumors than in E6-negative tumors (64.9% vs. 35.1%, *P* = 0.003). High Bcl-2 mRNA expression was more commonly occurred in low-miR-184 tumors than in high-miR-184 tumors (58.8% vs. 42.2%, *P* = 0.040). Bcl-2 protein expression evaluated by immunohistochemistry was negatively correlated with miR-184 expression in a small subset of tumors (*n* = 60, *P* = 0.038; Supplementary Table 1). However, miR-184 and Bcl-2 mRNA expression was not associated with clinical parameters in this study population including age, genders, cigarette smoking, and stages (Supplementary Table 2). These observations from patients' tumors seemed to support the action of mechanism in cell models to suggest that E6 oncoprotein may reduce miR-184 expression and, in turn, de-target Bcl-2 in lung tumors.

**Table 1:** Correlation of E6 with miR-184 and Bcl-2 expression and the relationship between miR- 184 and Bcl-2 mRNA levels in tumor tissues from NSCLC patients

Variable	Case No. (*n* = 136)	E6 expression				*P* value	miR-184				*P* value
		Negative (%)		Positive (%)			Low (%)		High (%)		
		79	(58.1)	57	(41.9)		68	(50.0)	68	(50.0)	
Bcl-2 mRNA											
Low	68	45	(66.2)	23	(33.8)	0.056	28	(41.2)	40	(58.8)	0.040
High	68	34	(50.0)	34	(50.0)		40	(58.8)	28	(41.2)	
miR-184											
Low	68	31	(39.2)	37	(64.9)	0.003					
High	68	48	(60.8)	20	(35.1)						
p53 mutation											
No	97						50	(51.5)	47	(48.5)	0.569
Yes	39						18	(46.2)	21	(53.8)	

E6-positive and -negative expression in lung tumors evaluated by immunohistochemistry obtained from our previous reports. MiR-184 and Bcl-2 mRNA levels in lung tumors were determined by real-time PCR analysis. The median value of miR-184 (0.98) and Bcl-2 mRNA (1.23) in this study population was used as a cutoff point to divide patients into “low” and “high” subgroup.

### Low miR-184, E6-positive, high Bcl-2 mRNA tumors, or both combinations are more commonly occurred unfavorable response to cisplatin-based chemotherapy

Fifty-nine out of 136 patients were available for the retrospective study to examine the possibility that E6, miR-184, Bcl-2, or both combinations may be associated with the tumor response to cisplatin-based chemotherapy in NSCLC. Patients with E6-positive, low-miR-184, and high-Bcl-2 tumors were more commonly occurred unfavorable response to chemotherapy than their counterparts (54.8% vs. 28.6%, *P* = 0.041 for E6, 52.8% vs. 26.1%, *P* = 0.043 for miR-184, 60.0% vs. 29.4%, *P* = 0.019 for Bcl-2; Table 2). Patients with E6-positive/low-miR-184, E6-positive/high-Bcl-2, or low-miR-184/high-Bcl-2 tumors were more frequently observed unfavorable response to chemotherapy than those with E6- negative/high-miR-184, E6-negative/low-Bcl-2, or high-miR-184/low-Bcl-2 tumors. However, the tumor response for the four categories of E6/miR-184 and E6/Bcl-2 did not reach the statistical significance (*P* = 0.075 for E6/miR-184, *P* = 0.070 for E6/Bcl-2); this could be due to a small number of patients in the category of E6- positive/high-miR-184 (*n* = 7) and E6-positive/low-Bcl-2 (*n* = 7) (Table 2). When patients were divided into two categories, patients with E6-positive/low-miR-184, E6-positive/high-Bcl-2, and low-miR-184/high-Bcl-2 tumors exhibited more prevalently occurred unfavorable response than the combination of three other categories (*P* = 0.010 for E6/miR-184 and E6/Bcl-2, *P* = 0.001 for miR- 184/Bcl-2; Table 2). These results suggest that E6- reduced miR-184 level may confer unfavorable response to cisplatin-based chemotherapy in NSCLC patients via increasing Bcl-2.

**Table 2:** Association of E6, miR-184 and Bcl-2 mRNA expression with tumor response to cisplatin-based chemotherapy in NSCLC patients

		Tumor Response				
	Case No. (*n* = 59)	Unfavorable (%)		Favorable (%)		*P*
**E6**						
Negative	28	8	(28.6)	20	(71.4)	0.041
Positive	31	20	(54.8)	14	(45.2)	
**miR-184**						
Low	36	19	(52.8)	17	(47.2)	0.043
High	23	6	(26.1)	17	(73.9)	
**Bcl-2**						
Low	34	10	(29.4)	24	(70.6)	0.019
High	25	15	(60.0)	10	(40.0)	
**E6/miR-184**						
Negative/High	16	4	(25.0)	12	(75.0)	0.075
Negative/Low	12	4	(33.3)	8	(66.7)	
Positive/High	7	2	(28.6)	5	(71.4)	
Positive/low	24	15	(62.5)	9	(37.5)	
Others	35	10	(28.6)	25	(71.4)	0.010
Positive/low	24	15	(62.5)	9	(37.5)	
**miR-184/Bcl-2**						
High/Low	19	6	(31.6)	13	(68.4)	0.006
Low/Low	15	4	(26.7)	11	(73.3)	
Low/High	21	15	(71.4)	6	(28.6)	
High/High	4	0	(0.0)	4	(100.0)	
Others	38	10	(26.3)	28	(73.7)	0.001
Low/High	21	15	(71.4)	6	(28.6)	
**E6/Bcl-2**						
Negative/Low	27	8	(29.6)	19	(70.4)	0.070
Negative/High	1	0	(0.0)	1	(100.0)	
Positive/low	7	2	(28.6)	5	(71.4)	
Positive/High	24	15	(62.5)	9	(37.5)	
Others	35	10	(28.6)	25	(71.4)	0.010
Positive/High	24	15	(62.5)	9	(37.5)	

The median value of miR-184 (0.98) and Bcl-2 mRNA (1.23) in this study population was used as a cutoff point to divide patients into “low” and “high” subgroup.

## DISCUSSION

Some miRs have been shown to confer drug resistance in various human cancers via targeting Bcl-2 [21]. For example, miR-204 targets Bcl-2 expression and enhances responsiveness of 5-fluorouracil and oxaliplatin in gastric cancer [22]. MiR-503 regulates the resistance to cisplatin in lung cancer by targeting Bcl-2 [23]. MiR- 24-2 expression may confer cisplatin sensitivity in breast cancer by targeting Bcl-2 [24]. Bcl-2 targeted by miR-184 promotes cell apoptosis in nasopharyngeal cancer [19]. We here provided evidence to support the previous report, indicating that a decrease in miR-184 by E6 oncoprotein confers cisplatin resistance in lung cancer cells and unfavorable chemotherapeutic response in NSCLC patients due to increasing Bcl-2 expression.

The colony formation assay was used to examine whether the colony formation efficacy reduced by cisplatin could be modulated by miR-184 manipulation. The colony formation efficacy in TL-1 cells with cisplatin treatment was significantly reduced by transfecting miR-184 mimic; however, the colony formation efficacy in TL-10 cells with cisplatin treatment was increased by transfecting miR-184 inhibitor when compared with their control cells (Supplementary Figure 1). These results from the colony formation assay in lung cancer cells with miR-184 manipulation were consistent with the observations of lung cancer patients, and suggest that a decrease in miR-184 expression may confer cisplatin resistance in lung cancer.

E6 expression was negatively correlated with miR- 184 expression, but p53 mutation status was not associated with miR-184 expression in tumor tissues from lung cancer patients (*P* = 0.569; Table 1). This conflicting might be due to wild-type p53 dysfunction by E6 oncoprotein in lung cancer patients [10]. In addition, reduced miR-184 expression by E6 oncoprotein has been evidenced by the cell models (Figure 1). A higher prevalence of unfavorable response to cisplatin-based chemotherapy in patients with E6-positive, low-miR-184 and high-Bcl-2 mRNA tumors compared with their counterparts was observed in this study population (Table 2). In addition, the highest frequency of unfavorable response to cisplatin-based chemotherapy was revealed in patients with E6-positive/low-miR-184, low-miR-184/high-Bcl-2, E6-positive/high-Bcl-2 tumors among their four categories (Table 2). These results from the cell model and human tissues support the hypothesis that a decrease in miR-184 by E6 oncoprotein may play more important role than p53 mutation status on the elevation of Bcl-2 expression and consequently to confer cisplatin resistance and unfavorable response to cisplatin-based chemotherapy in NSCLC.

P53 pathway has been shown to modulate chemosensitivity in human cancer [25, 26]. For example, inactivation of p53 pathway by HPV infections may confer chemoresistance in cervical cancer via increasing Bcl-2 and Mcl-1 expressions [27, 28]. Our previous study indicated that upregulation of cIAP2 by E6 oncoprotein via EGFR/PI3K/AKT pathway confers cisplatin resistance in HPV-infected lung cancer [29]. In the current study, the evidence from the cell model strongly supported that miR- 184 down-regulated by E6 oncoprotein via inactivation of p53 pathway conferred cisplatin resistance due to Bcl-2 de- targeted by E6-mediated miR-184 reduction (Figures 1–5). Moreover, Bcl-2 de-targeted by miR-184 reduction may be responsible for E6-mediated cisplatin resistance in cervical and lung cancer cells (Figure 6). To the best our knowledge, this is the first study revealed that a decrease in miR-184 by E6-mediated p53 degradation may confer cisplatin-based chemotherapy in HPV-associated human cancers including cervical and lung cancer.

The elevation of Bcl-2 expression by paxillin and MnSOD conferred cisplatin resistance and unfavorable response to cisplatin-based chemotherapy in NSCLC [30, 31]. Bcl-2 antagonist ABT-199 has been shown to cause on-target cell death in acute myeloid leukemia, and significantly enhances imatinib-induced cell death in chronic myeloid leukemia [32, 33]. MnSOD-induced cisplatin resistance can be overcome by ABT-199 in lung cancer cells and xenograft tumors [30]. We further provided evidence that ABT-199 can overcome E6-induced cisplatin resistance in TL-1 and SiHa cells when compared with their NC cells (8.9 vs. 22.2 for TL-1, 10.7 vs. 25.1 for SiHa); however, the overcome of ABT-199 in cisplatin resistance did not observe in E6-knockdown both cell types (Supplementary Figure 2 upper panel). Similar findings in ABT-199 against E6-induced cisplatin resistance were also revealed in E6-overexpressing TL-10 and C33A cells (Supplementary Figure 2 lower panel). We therefore suggest that Bcl-2 antagonist might potentially overcome cisplatin resistance mediated by E6-reduced miR-184, and consequently to improve outcomes in NSCLC patients with E6-positive/low-miR-184 tumors.

## MATERIALS AND METHODS

### Study subjects

Lung tumor specimens were collected from 136 patients with primary NSCLC surgical resection at the Department of Thoracic Surgery, Taichung Veterans General Hospital (Taichung, Taiwan) between 1998 and 2004. Patients were asked to submit written informed consent; the study was approved by the Institutional Review Board (TMUH No. 201301051). The tumor type and stage of each collected specimen were histologically determined in accordance with the World Health Organization classification system. Cancer relapse data were obtained from chart review and confirmed by thoracic surgeons. Clinical parameters and OS and RFS data were collected from chart review and the Taiwan Cancer Registry, Department of Health, Executive Yuan, ROC.

### Cell lines

TL-1 cells were kindly provided by Dr. Y.-W. Cheng (Graduate Institute of Cancer Biology and Drug Discovery, Taipei Medical University, Taipei, Taiwan) [10]. TL-10 cells were primarily cultured from pleural effusions of lung adenocarcinoma patients by our laboratory. SiHa and C33A cells were obtained from the Bioresource Collection and Research Center, the Food Industry Research and Development Institute (Hsinchu, Taiwan). TL-1, TL-10 and C33A cancer cell lines were maintained in RPMI-1640 (HyClone, Logan, UT). SiHa cancer cell lines were maintained in DMEM (HyClone, Logan, UT). The medium contained 10% fetal bovine serum (FBS) supplemented with penicillin (100 U/mL) and streptomycin (100 mg/mL). These cells were cultured in accordance with the suppliers' instructions. Once resuscitated, cell lines were routinely authenticated (once every 6 months; the cells were last tested in December 2012) by means of cell morphology monitoring, growth curve analysis, species verification via isoenzymology and karyotyping, identity verification via short tandem repeat profiling analysis, and contamination checks.

### Plasmid construction, transfection, and stable clone selection

The wild-type and mutant p53 constructs were kindly provided by Dr. Jiunn-Liang Ko (Institute of Medicine, Chung Shan Medical University, Taichung, Taiwan). HPV 16 E6 and HPV 18 E6 cDNA and HPV 16 E6 and HPV 18 E6 shRNA were as previously described [7]. Delete miR-184 promoter constructs were constructed by the QuickChange site-directed mutagenesis system (Stratagene, La Jolla, CA). The Bcl-2-Flag overexpression plasmid constructed into pCMV-Tag2B vector was purchased from Addgene (Addgene Company, Cambridge, MA). The p53 and Bcl-2 shRNA were obtained from the National RNAi Core Facility, Academia Sinica provided. The target sequences for shRNA are presented in Supplementary Table 3. Nonspecific shRNA of the scramble sequence was used as the control in the knockdown experiment, and an empty vector expression was used as the control for the overexpression plasmid. The transfection and stable clone selection procedures have been described previously [34].

### The mimic and inhibitor of miR-184 transfection

MicroRNA mimic (pre-miR-184, 20–40 nmol/L/well; Ambion, Foster City, CA), MicroRNA inhibitor (20– 40 nmol/L/well; Ambion, Foster City, CA), and negative control (Ambion, Foster City, CA) were transfected using Lipofectamine 3000 transfection reagent (Invitrogen, Foster City, CA) according to the manufacturers' protocols. Transfection efficiency was evaluated by a real-time polymerase chain reaction (PCR).

### Real-time PCR analysis

DNase I–treated total RNA (10 ng) was subjected to miRNA RT-PCR analysis with the TaqMan miRNA Reverse Transcription Kit (Applied Biosystems, Foster City, CA), miRNA Assays (Applied Biosystems, Foster City, CA), and a Real-Time Thermocycler 7500 (Applied Biosystems, Foster City, CA). RNU6B was used as the microRNA reference housekeeping gene, and GAPDH was used as the mRNA reference housekeeping gene. The primers used for real-time PCR analysis of mRNA expression are presented in Supplementary Table 3. The mRNA and microRNA levels in tumours that were higher than the median value were defined as “high”, whereas levels lower than the median value were defined as “low”.

### Luciferase reporter assay

Double-stranded oligonucleotides corresponding to the wild-type or delete p53 binding site in the promoter region of miR-184 were synthesized and ligated between the SpeI and HindIII restriction sites of pGL3 Reporter Vector (Ambion, Foster City, CA). The oligonucleotides utilized are listed in Supplementary Table 3. Cells were transfected using an appropriate plasmid. Luciferase assays were done with the luciferase reporter assay system (Promega, Fitchburg, WI) 48 h after transfection. Normalized luciferase activity was reported as luciferase activity/β-galactosidase activity.

### ChIP assay

ChIP analysis was performed as described previously [35]. The primer sequences are presented in Supplementary Table 3.

### 3-(4,5-cimethylthiazol-2-yl)-2,5-diphenyl tetrazolium bromide (MTT) cytotoxicity assay

The cell lines were cultured in 96-well flat-bottomed microtiter plates supplemented with RPMI 1640 and DMEM containing 10% heat-inactivated fetal bovine serum, 100 units/mL penicillin, and 100 units/mL streptomycin in a humidified atmosphere containing 95% air and 5% CO_2_ at 37°C in a humidified incubator. Before cisplatin treatment (0, 2, 4, 8, 16, 32 μM), the cells cultured in the exponential growth phase were pretreated with miR mimic, inhibitor, shRNAs, p53 and Bcl-2 overexpression plasmid for 24 h. After 48 h incubation, the *in vitro* cytotoxic effects of these treatments were determined by MTT assay (at 570 nm).

### Statistical analysis

All statistical analyses were conducted using the SPSS statistical software program as described previously (version 17.0; SPSS, Inc., Chicago, IL) [34, 35]. A two-sided analysis of the variance in the statistical tests was conducted, and *P* values < 0.050 were considered statistically significant.

## SUPPLEMENTARY MATERIALS FIGURES AND TABLES



## References

[1] Cheng Z, Wang HZ, Li X, Wu Z, Han Y, Li Y, Chen G, Xie X, Huang Y, Du Z, Zhou Y (2015). MicroRNA-184 inhibits cell proliferation and invasion, and specifically targets TNFAIP2 in Glioma. J Exp Clin Cancer Res.

[2] Foley NH, Bray IM, Tivnan A, Bryan K, Murphy DM, Buckley PG, Ryan J, O'Meara A, O'sullivan M, Stallings RL (2010). MicroRNA-184 inhibits neuroblastoma cell survival through targeting the serine/threonine kinase AKT2. Mol Cancer.

[3] Wong TS, Liu XB, Wong BY, Ng RW, Yuen AP, Wei WI (2008). Mature miR-184 as Potential Oncogenic microRNA of Squamous Cell Carcinoma of Tongue. Clin Cancer Res.

[4] Liu Z, Mai C, Yang H, Zhen Y, Yu X, Hua S, Wu Q, Jiang Q, Zhang Y, Song X, Fang W (2014). Candidate tumour suppressor CCDC19 regulates miR-184 direct targeting of C-Myc thereby suppressing cell growth in non-small cell lung cancers. J Cell Mol Med.

[5] Lin TC, Lin PL, Cheng YW, Wu TC, Chou MC, Chen CY, Lee H (2015). MicroRNA-184 Deregulated by the MicroRNA-21 Promotes Tumor Malignancy and Poor Outcomes in Non-small Cell Lung Cancer via Targeting CDC25A and c-Myc. Ann Surg Oncol.

[6] Cheng YW, Wu TC, Chen CY, Chou MC, Ko JL, Lee H (2008). Human telomerase reverse transcriptase activated by E6 oncoprotein is required for human papillomavirus-16/18-infected lung tumorigenesis. Clin Cancer Res.

[7] Sung WW, Wang YC, Lin PL, Cheng YW, Chen CY, Wu TC, Lee H (2013). IL-10 promotes tumor aggressiveness via upregulation of CIP2A transcription in lung adenocarcinoma. Clin Cancer Res.

[8] Wu DW, Tsai LH, Chen PM, Lee MC, Wang L, Chen CY, Cheng YW, Lee H (2012). Loss of TIMP-3 promotes tumor invasion via elevated IL-6 production and predicts poor survival and relapse in HPV-infected non-small cell lung cancer. Am J Pathol.

[9] Chen PM, Cheng YW, Wang YC, Wu TC, Chen CY, Lee H (2014). Up-regulation of FOXM1 by E6 oncoprotein through the MZF1/NKX2-1 axis is required for human papillomavirus-associated tumorigenesis. Neoplasia.

[10] Cheng YW, Wu MF, Wang J, Yeh KT, Goan YG, Chiou HL, Chen CY, Lee H (2007). Human papillomavirus 16/18 E6 oncoprotein is expressed in lung cancer and related with p53 inactivation. Cancer Res.

[11] Cheng YW, Chiou HL, Sheu GT, Hsieh LL, Chen JT, Chen CY, Su JM, Lee H (2001). The association of human papillomavirus 16/18 infection with lung cancer among nonsmoking Taiwanese women. Cancer Res.

[12] Fei Y, Yang J, Hsieh WC, Wu JY, Wu TC, Goan YG, Lee H, Cheng YW (2006). Different human papillomavirus 16/18 infection in Chinese non-small cell lung cancer patients living in Wuhan, China. Jpn J Clin Oncol.

[13] Ragin C, Obikoya-Malomo M, Kim S, Chen Z, Flores-Obando R, Gibbs D, Koriyama C, Aguayo F, Koshiol J, Caporaso NE, Carpagnano GE, Ciotti M, Dosaka-Akita H (2014). HPV-associated lung cancers: an international pooled analysis. Carcinogenesis.

[14] Li YJ, Tsai YC, Chen YC, Christiani DC (2009). Human papilloma virus and female lung adenocarcinoma. Semin Oncol.

[15] Anantharaman D, Gheit T, Waterboer T, Halec G, Carreira C, Abedi-Ardekani B, McKay-Chopin S, Zaridze D, Mukeria A, Szeszenia-Dabrowska N, Lissowska J, Mates D, Janout V (2014). No causal association identified for human papillomavirus infections in lung cancer. Cancer Res.

[16] Hockenbery DM, Oltvai ZN, Yin XM, Milliman CL, Korsmeyer SJ (1993). Bcl-2 functions in an antioxidant pathway to prevent apoptosis. Cell.

[17] Yang J, Liu X, Bhalla K, Kim CN, Ibrado AM, Cai J, Peng TI, Jones DP, Wang X (1997). Prevention of apoptosis by Bcl-2: release of cytochrome c from mitochondria blocked. Science.

[18] Kroemer G (1997). The proto-oncogene Bcl-2 and its role in regulating apoptosis. Nat Med.

[19] Zhen Y, Liu Z, Yang H, Yu X, Wu Q, Hua S, Long X, Jiang Q, Song Y, Cheng C, Wang H, Zhao M, Fu Q (2013). Tumor suppressor PDCD4 modulates miR-184-mediated direct suppression of C-MYC and BCL2 blocking cell growth and survival in nasopharyngeal carcinoma. Cell Death Dis.

[20] Wang DT, Ma ZL, Li YL, Wang YQ, Zhao BT, Wei JL, Qi X, Zhao XT, Jin YX (2013). miR-150, p53 protein and relevant miRNAs consist of a regulatory network in NSCLC tumorigenesis. Oncol Rep.

[21] Wang Y, Lee CG (2009). MicroRNA and cancer—focus on apoptosis. J Cell Mol Med.

[22] Sacconi A, Biagioni F, Canu V, Mori F, Di Benedetto A, Lorenzon L, Ercolani C, Di Agostino S, Cambria AM, Germoni S, Grasso G, Blandino R, Panebianco V (2012). miR-204 targets Bcl-2 expression and enhances responsiveness of gastric cancer. Cell Death Dis.

[23] Qiu T, Zhou L, Wang T, Xu J, Wang J, Chen W, Zhou X, Huang Z, Zhu W, Shu Y, Liu P (2013). miR-503 regulates the resistance of non-small cell lung cancer cells to cisplatin by targeting Bcl-2. Int J Mol Med.

[24] Srivastava N, Manvati S, Srivastava A, Pal R, Kalaiarasan P, Chattopadhyay S, Gochhait S, Dua R, Bamezai RN (2011). miR- 24-2 controls H2AFX expression regardless of gene copy number alteration and induces apoptosis by targeting antiapoptotic gene BCL-2: a potential for therapeutic intervention. Breast Cancer Res.

[25] Wu GS, El-Diery WS (1996). p53 and chemosensitivity. Nat Med.

[26] El-Deiry WS (2003). The role of p53 in chemosensitivity and radiosensitivity. Oncogene.

[27] Leisching G, Loos B, Botha M, Engelbrecht AM (2015). Bcl-2 confers survival in cisplatin treated cervical cancer cells: circumventing cisplatin dose-dependent toxicity and resistance. J Transl Med.

[28] Wei LH, Kuo ML, Chen CA, Chou CH, Cheng WF, Chang MC, Su JL, Hsieh CY (2001). The anti-apoptotic role of interleukin-6 in human cervical cancer is mediated by up-regulation of Mcl-1 through a PI 3-K/Akt pathway. Oncogene.

[29] Wu HH, Wu JY, Cheng YW, Chen CY, Lee MC, Goan YG, Lee H (2010). cIAP2 upregulated by E6 oncoprotein via epidermal growth factor receptor/phosphatidylinositol 3-kinase/AKT pathway confers resistance to cisplatin in human papillomavirus 16/18-infected lung cancer. Clin Cancer Res.

[30] Chen PM, Cheng YW, Wu TC, Chen CY, Lee H (2015). MnSOD overexpression confers cisplatin resistance in lung adenocarcinoma via the NF-kappaB/Snail/Bcl-2 pathway. Free Radic Biol Med.

[31] Wu DW, Wu TC, Wu JY, Cheng YW, Chen YC, Lee MC, Chen CY, Lee H (2014). Phosphorylation of paxillin confers cisplatin resistance in non-small cell lung cancer via activating ERK-mediated Bcl-2 expression. Oncogene.

[32] Ko TK, Chuah CT, Huang JW, Ng KP, Ong ST (2014). The BCL2 inhibitor ABT-199 significantly enhances imatinib-induced cell death in chronic myeloid leukemia progenitors. Oncotarget.

[33] Pan R, Hogdal LJ, Benito JM, Bucci D, Han L, Borthakur G, Cortes J, DeAngelo DJ, Debose L, Mu H, Dohner H, Gaidzik VI, Galinsky I (2014). Selective BCL-2 inhibition by ABT-199 causes on-target cell death in acute myeloid leukemia. Cancer Discov.

[34] Sung WW, Wang YC, Cheng YW, Lee MC, Yeh KT, Wang L, Wang J, Chen CY, Lee H (2011). A polymorphic -844T/C in FasL promoter predicts survival and relapse in non-small cell lung cancer. Clin Cancer Res.

[35] Wu DW, Liu WS, Wang J, Chen CY, Cheng YW, Lee H (2011). Reduced p21(WAF1/CIP1) via alteration of p53-DDX3 pathway is associated with poor relapse-free survival in early-stage human papillomavirus-associated lung cancer. Clin Cancer Res.

