# Supplementary material for: Reduction of microRNA-184 by E6 oncoprotein confers cisplatin resistance in lung cancer via increasing Bcl-2
Source: Oncotarget. 2016 Apr 12;7(22):32362–74. doi: 10.18632/oncotarget.8708 (PMC5078019; doi:10.18632/oncotarget.8708)
Supplement: Supplementary file 1 [file oncotarget-07-32362-s001.pdf]

Reduction of microRNA-184 by E6 oncoprotein confers cisplatin resistance in lung cancer via increasing Bcl-2

Supplementary Materials

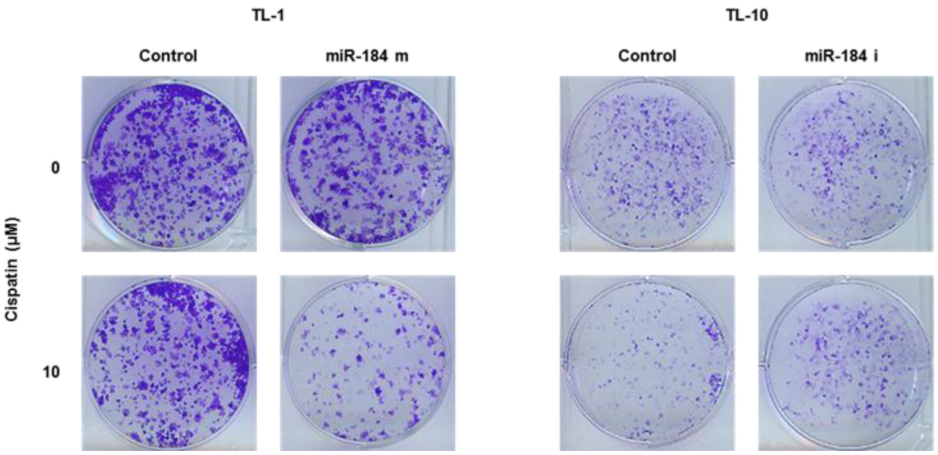

Supplementary Figure S1: Effects of miR-184 on the colony formation efficacy in TL-1 and TL-10 cells. TL-1 and TL-10 cells were respectively transfected with miR-184 mimic and inhibitor (10 μM) and the colony formation assay was performed for 14 days to examine the effects of miR-184 manipulation on the efficacy of the colony formation on agar plates.

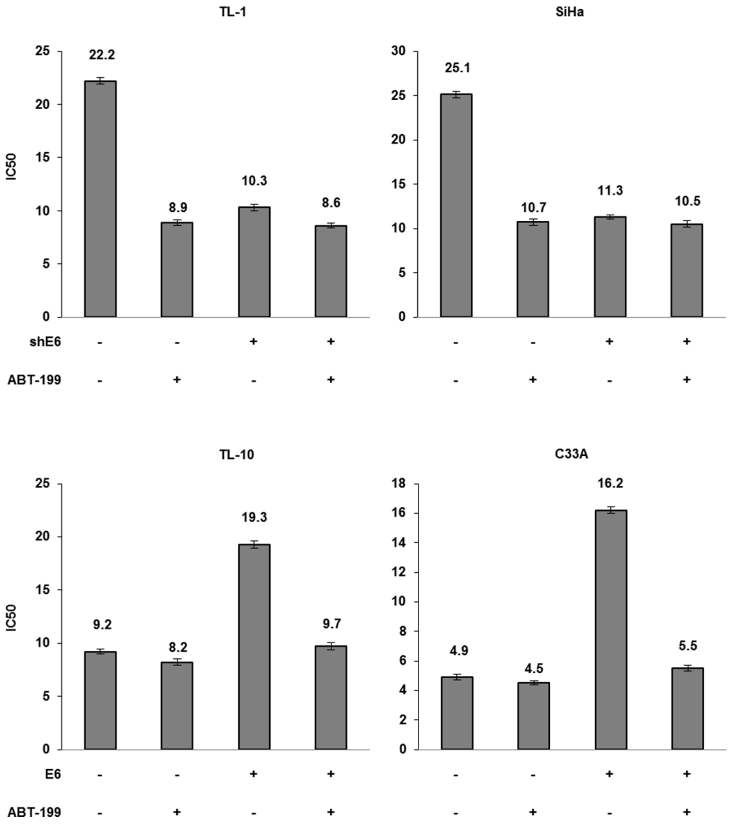

Supplementary Figure S2: ABT-199 can overcome E6-induced cisplatin resistance. shE6 plasmids were transfected into E6-positive cell lines (TL-1 and SiHa) compared with both cell types transfected with a non-specific shRNA (NC), E6 expression vector were transfected into E6 negative (TL-10 and C33A) cell lines compared with both cell types transfected with an empty vector (VC). After 24 h, the indicated cells were incubated with or without ABT-199 (0.3 μM) and cisplatin (0, 2, 4, 8, 16, 32 μM) for 48 h and then the change of IC50 value was determined by the MTT assay.

Supplementary Table S1: The association of miR-184 levels with Bcl-2 mRNA and protein expressions in NSCLC patients

Characteristics	Patient No.	miR-184				P value
		Low (%)		High (%)		
**Total patients**	60	28	(46.7)	32	(53.3)	
**Bcl-2 protein**						
Negative	30	10	(33.3)	20	(66.7)	0.038
Positive	30	18	(60.0)	12	(40.0)	

Supplementary Table S2: Relationships of miR-184 and Bcl-2 mRNA levels with clinical parameters in NSCLC patients

Parameters	Case No. (n = 136)	miR-184				*P*	Bcl-2 mRNA				*P*
		Low (%)		High (%)			Low (%)		High (%)		
**Age**											
≤ 65	66	32	(48.5)	34	(51.5)	0.731	36	(54.5)	30	(45.5)	0.303
> 65	70	36	(51.4)	34	(48.6)		32	(45.7)	38	(54.3)	
**Gender**											
Female	47	21	(44.7)	26	(55.3)	0.367	25	(53.2)	22	(46.8)	0.589
Male	89	47	(54.8)	42	(47.2)		43	(48.3)	46	(51.7)	
**Smoking**											
Nonsmoker	77	41	(53.2)	36	(46.8)	0.387	39	(50.6)	38	(49.4)	0.863
Smoker	59	27	(45.8)	32	(54.2)		29	(49.2)	30	(50.8)	
**Stage**											
I	45	24	(53.3)	21	(46.7)	0.218	23	(51.1)	22	(48.9)	0.981
II	26	9	(34.6)	17	(65.4)		13	(50.0)	13	(50.0)	
III	65	35	(53.8)	30	(46.2)		32	(49.2)	33	(50.8)	

The median value of miR-184 (0.98) and Bcl-2 mRNA (1.23) in this study population were used as a cutoff point to divide patients into "low" and "high" subgroup.

Supplementary Table S3: List of primer sequences used in the present study

Target gene	Sequence
**miR184 promoter reporter plasmid**	
Forward	5′- GGTACCCAAGCAGATGGGATCCAAAGTTGGTG -3′
Reverse	5′- CTCGAGGGCAAAGAAGTTTTGATGAAGCTTTG -3′
Mutated p53 binding site Forward	5′-GGCGAGCCCTCCCCTCTCTGCAGT-3′
Mutated p53 binding site Reverse	5′-ACTGCAGAGAGGGGAGGGCTCGCC-3′
**ChIP primer**	
Forward	5′- ACCTATTACTTTCCATAGCTGTCCA -3′
Reverse	5′- CTGGCCGGCAAAGAAGTTTT-3′
**RNAi target**	
Shp53	5′- CACCATCCACTACAACTACAT-3′
shBcl-2	5′- TGGATGACTGAGTACCTGAAC-3′
